# Supplementary figures and images for: A coupling model of transcranial magnetic stimulation induced electric fields to neural state variables
Source: PLoS Comput Biol. 2026 Mar 20;22(3):e1013413. doi: 10.1371/journal.pcbi.1013413 (PMC13038122; doi:10.1371/journal.pcbi.1013413)

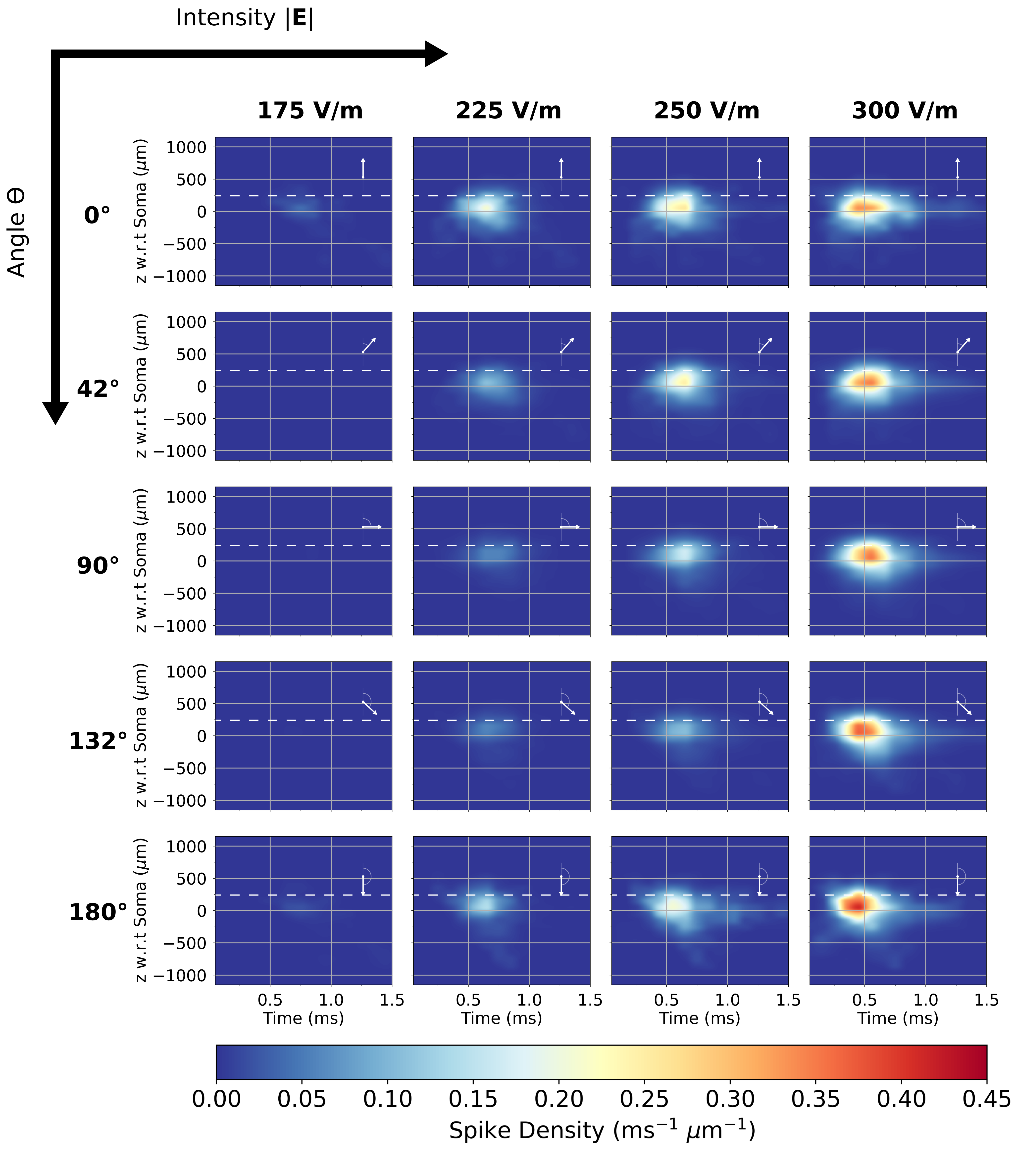

Supplement: S1 Fig — Kernels are shown in a grid with electric field intensity |E| increasing to the right and polar angle θ increasing to the bottom (depiction of electric field orientation w.r.t somatodendritic axis overlaid top right of each kernel). Each kernel is plotted with respect to time and the depth with respect to cell soma. The color scale is spike density. (TIF) [file pcbi.1013413.s002.tif]

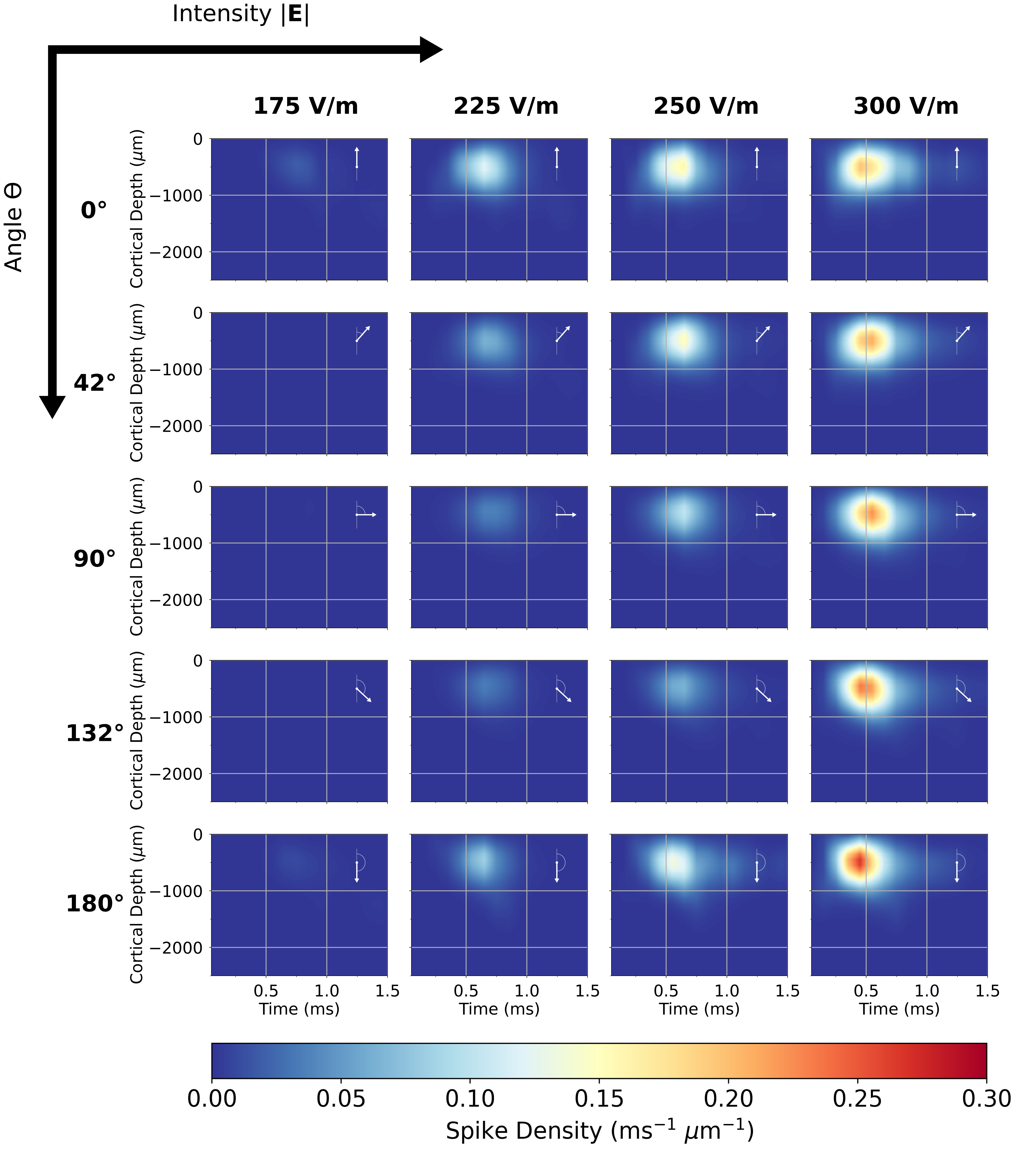

Supplement: S2 Fig — (S1 Fig after correlation with the L2/3 cell density function from Fig 5D and scaling by pre-:post-synaptic cell count ratio). Kernels are shown in a grid with electric field intensity |E| increasing to the right and polar angle θ increasing to the bottom (depiction of electric field orientation w.r.t somatodendritic axis overlaid top right of each kernel). Each kernel is plotted w.r.t. time and cortical depth from the CSF boundary. The color scale is spike density. (TIF) [file pcbi.1013413.s003.tif]

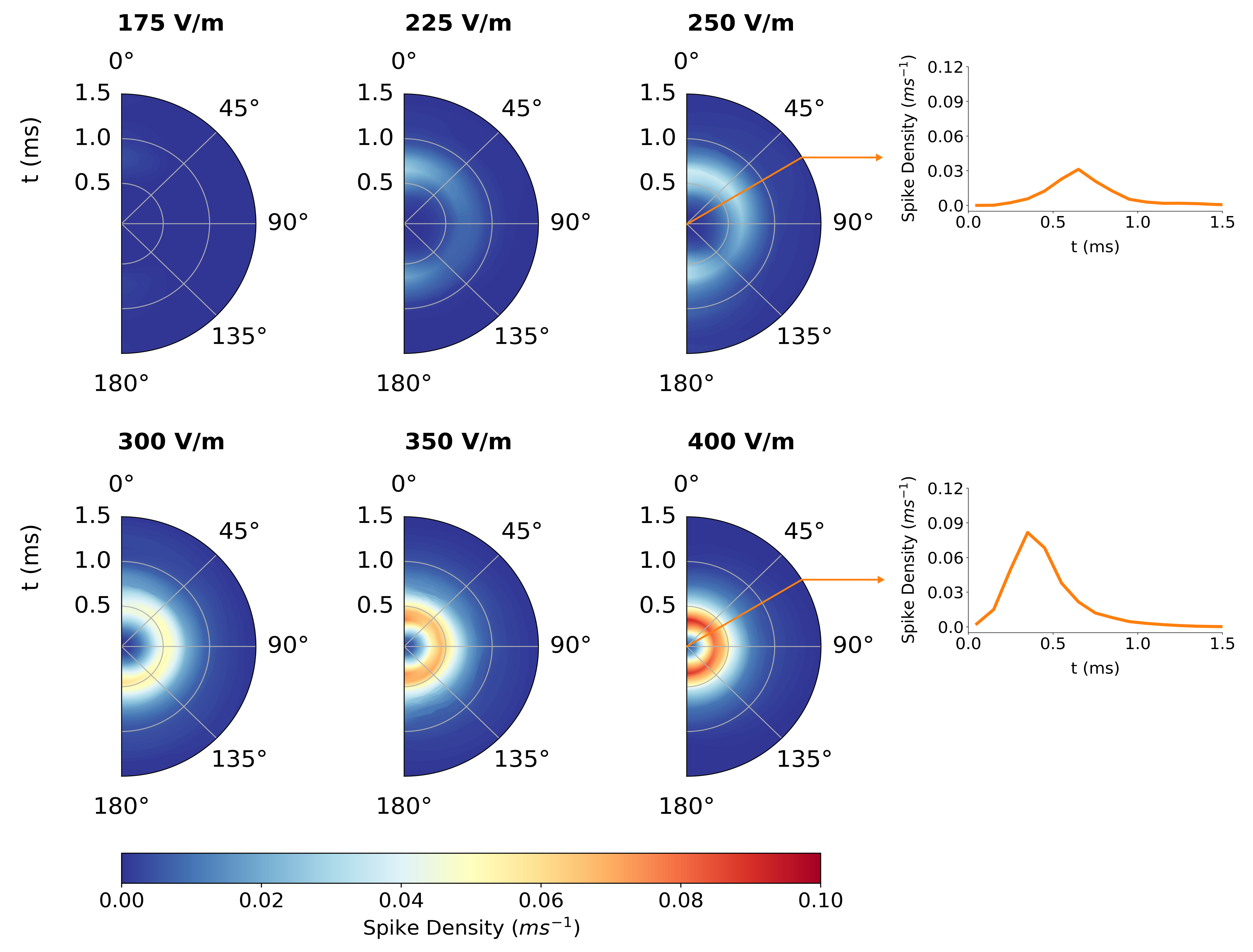

Supplement: S3 Fig — Half polar plots shown for six electric field intensity |E| values. 2D kernels from S2 Fig are averaged across z to produce a time-dependent curve for each angle. The radial axis of each polar plot is time, the polar axis is the angle, θ, the electric field vector makes with the somatodendritic axis, and the color scale is partial spike density. Top right and bottom right are crosscuts of the |E|=250 V/m and 400 V/m polar plots for θ= 60°. (TIF) [file pcbi.1013413.s004.tif]

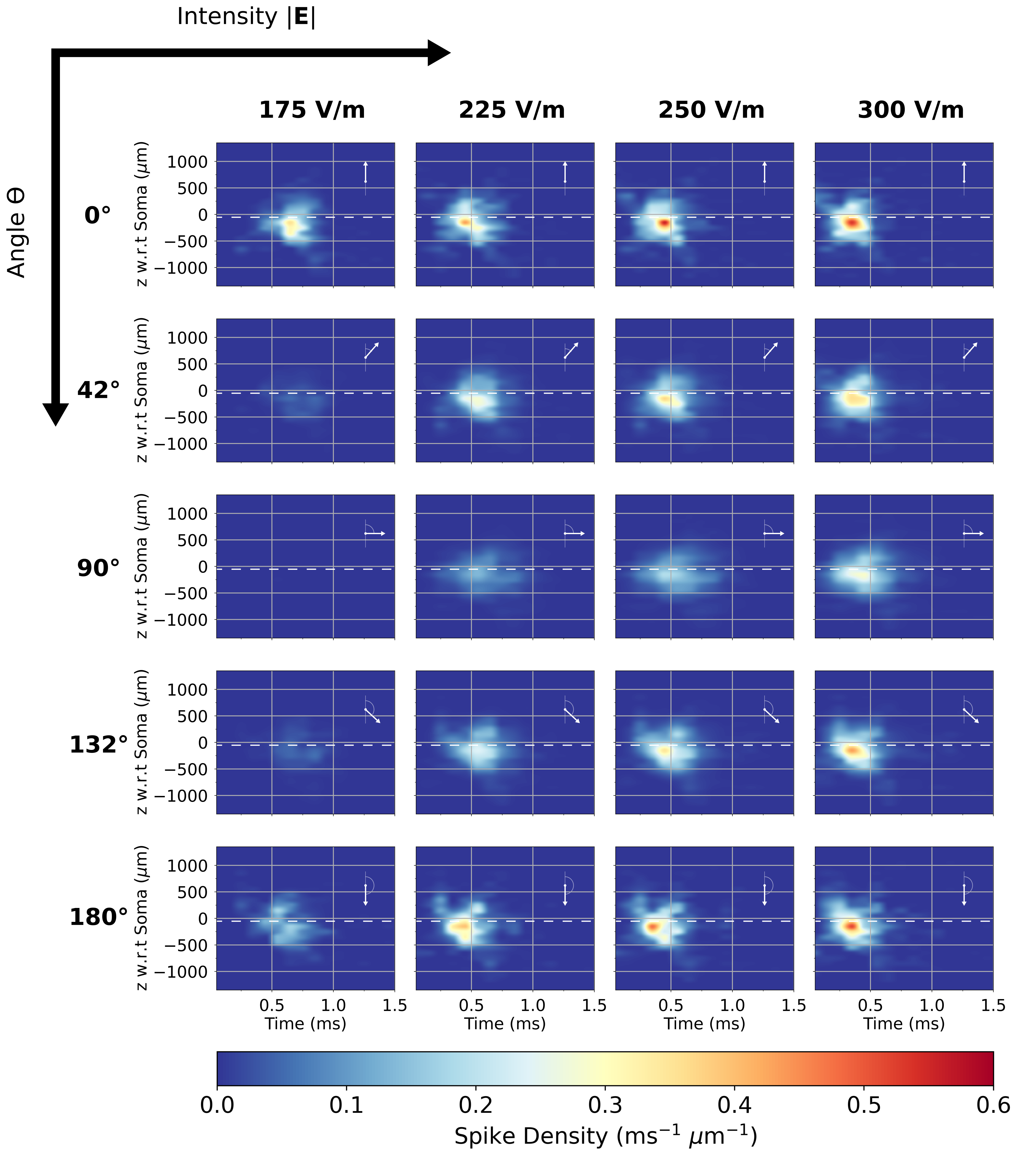

Supplement: S4 Fig — Kernels are shown in a grid with electric field intensity |E| increasing to the right and polar angle θ increasing to the bottom (depiction of electric field orientation w.r.t somatodendritic axis overlaid top right of each kernel). Each kernel is plotted with respect to time and the depth with respect to cell soma. The color scale is spike density. (TIF) [file pcbi.1013413.s005.tif]

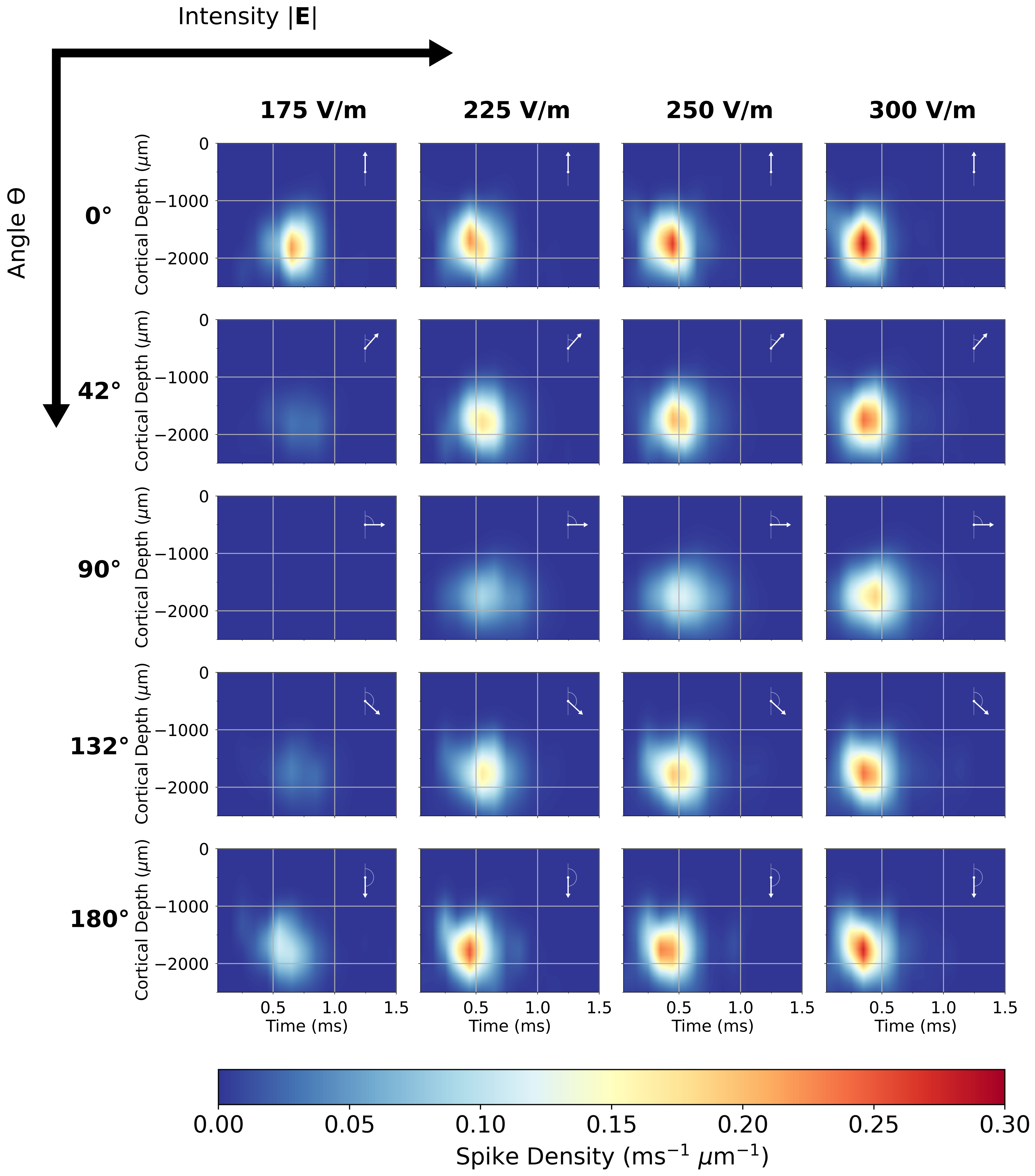

Supplement: S5 Fig — Kernels are shown in a grid with electric field intensity |E| increasing to the right and polar angle θ increasing to the bottom. Each kernel is plotted with respect to time and the cortical depth with respect to CSF boundary. The color scale is spike density. (TIF) [file pcbi.1013413.s006.tif]

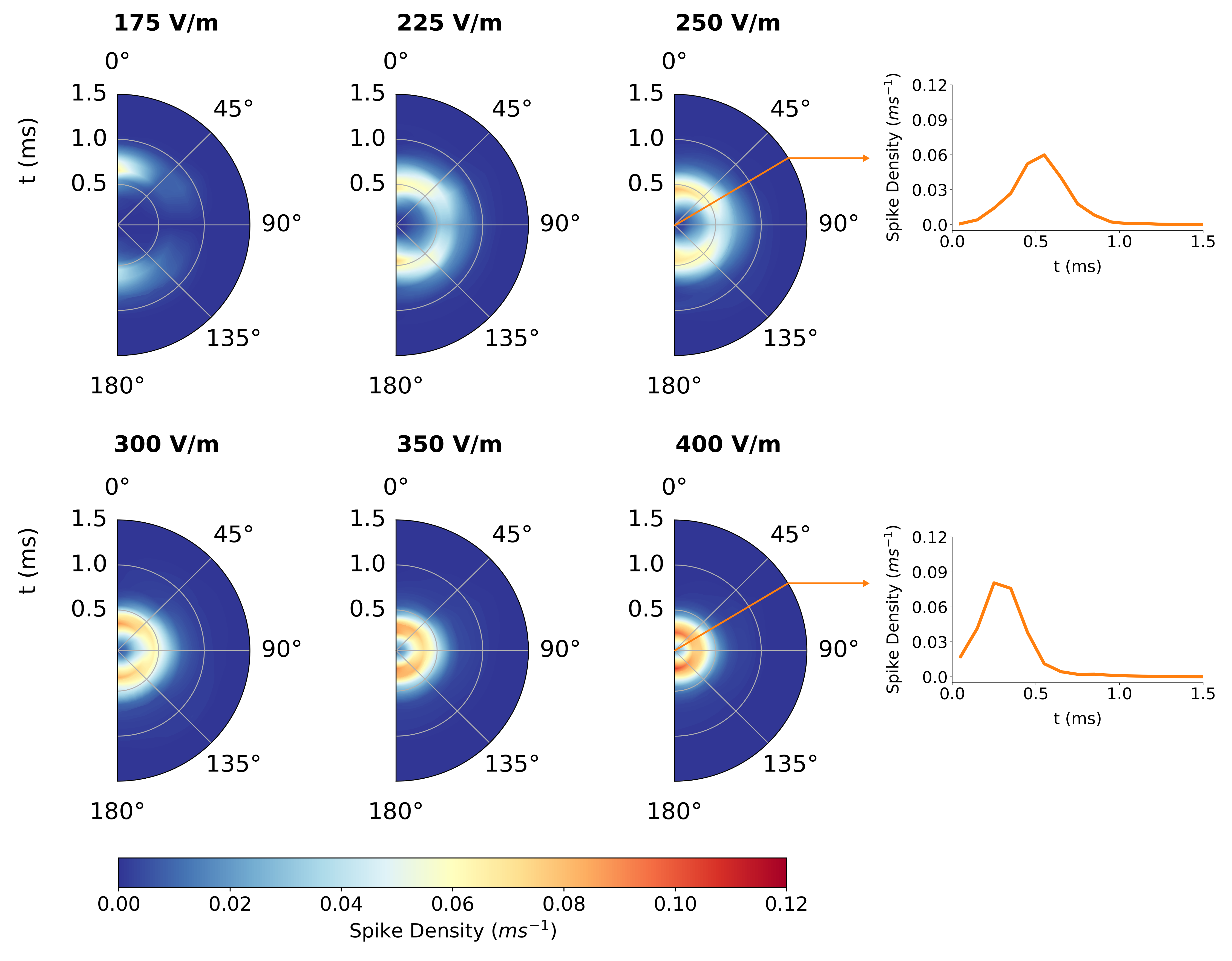

Supplement: S6 Fig — 2D kernels from S5 Fig are averaged across z to produce a time-dependent curve for each angle. The radial axis of each polar plot is time, the polar axis is the angle θ the electric field vector makes with the somatodendritic axis, and the color scale is partial spike density. Top left is depicted a crosscut for |E|=250 V/m, θ= 60°. (TIF) [file pcbi.1013413.s007.tif]
