# Supplementary material for: A coupling model of transcranial magnetic stimulation induced electric fields to neural state variables
Source: PLoS Comput Biol. 2026 Mar 20;22(3):e1013413. doi: 10.1371/journal.pcbi.1013413 (PMC13038122; doi:10.1371/journal.pcbi.1013413)
Supplement: Attachment — Submitted filename: response_to_reviewers.pdf [file pcbi.1013413.s008.pdf]

Responses to the reviewers' comments

Please find below the list of reviewer comments in order as they appear in the review letter. All text from the reviewers is in regular text, labeled Reviewer #1 and Reviewer #2. All responses by the authors are in italics, with line numbers and any altered text snippets copied below, each referring to the marked up copy of the manuscript. Updated lines in the manuscript are rendered in blue color.

Reviewer #1 Summary:

Reviewer #1: In this paper, the authors develop a pipeline for simulating the effects of transcranial magnetic stimulation (TMS) onto a postsynaptic population of cells. In particular, they consider the "indirect" effect of TMS on layer 5 (L5) pyramidal cells receiving inputs from L2/3 pyramidal and basket cells, which in turn are directly stimulated by TMS via activation of the axonal terminals that are aligned to the direction of the electric field generated by TMS stimulation. The paper is clear and the results that it presents are interesting: however, I think some changes should be made before the paper is suitable for publication in PLOS Computational Biology.

I have two main concerns about this paper, which are described in the following.

Reviewer #1: My first main observation has to do with the expectations that the authors build in the introduction, and that remain largely unmet at the end of the paper. In particular, in the introduction the authors describe the state of the art of TMS as it is used in a clinical setting, and present the full motor TMS cascade in Fig. 1: however, they then focus specifically on just one aspect of the full cascade, namely coupling the electrical field to the activation of neurons in L2/3 and L5 of the cortex. While there is nothing wrong with this (I realize that dealing with the whole pipeline as shown in Fig. 1 is well beyond the scope of pretty much any paper), and the authors clearly state it in the introduction, I think it would be beneficial if they toned down their claims and explained in more detail what the actual purpose of this paper is.

Response: As the reviewer correctly states, we first painted the bigger picture in order to provide proper motivation for our work, and then narrowed it down to one important aspect of the entire modeling chain, namely the coupling of the electric field to neuronal activation. We agree that this narrative could be initially misleading.
In order to prevent readers from being caught in too high expectations and then disappointed, we now applied changes to the first two paragraphs of the introduction, which are intended to make the actual scope of the paper more explicit up-front:

Line 46 - 55
"Non-invasive brain stimulation is a powerful set of tools that can modulate brain activity through the safe application of electromagnetic fields and without the need for surgery or implants. TMS offers targeted modulation of neural activity giving rise to immediate and long-lasting effects, making it valuable for probing and influencing the nervous system for diagnostic, therapeutic, and scientific purposes. However, in order to draw accurate conclusions about TMS activation and behavioral effects, we must first understand the mechanistic relationship between stimulation and observable readouts. While modeling approaches may entail variable chains of computation steps, depending on the stimulated brain area and the considered readout, they all rely on one crucial ingredient, namely the precise way the induced electric field excites populations of interconnected neurons. In this study, we develop a generic computational technique to tackle this step and study it using the example of TMS of the primary motor cortex (M1).

M1-TMS is widely studied through behavioral experiments and measurements of electrophysiological readouts. Experimental measurements of the brain, spinal cord, and activated muscles enable the study of structure-function relationships in the motor system as well as pre-surgical planning and clinical diagnostics. The mechanism between TMS stimulation of M1 and muscle contraction is a complex cascade …"

Reviewer #1: The second concern I have regards the practical applicability of the proposed pipeline: while the method looks intriguing, I don't think the authors do a good job of explaining why it is superior (or alternative) to other techniques such as the ones described, for instance, in [1] and [2]. In the latter reference, in particular, the authors explicitly model D+ and D- responses and compare their results to experiments, something that would reside in panel D of Fig. 1 of the present manuscript. In order to be relevant, I think that new results should be an improvement over the current state of the art, rather than another way of doing something that might lead to potentially interesting results.

Response: We acknowledge that we did not sufficiently clarify the relation between our framework and existing modeling approaches. We would like to emphasize that our contribution does not form a radically different approach, but rather offers a framework for adding important aspects to existing schemes. In particular, it adds directional/electric field sensitivity which has generally previously been ignored, and it provides an efficient way to summarize the effective architectures of axo-dendritic compounds within neuronal populations with respect to excitability.

- *Compared to Aberra and colleagues (2020), our model compacts the results of numerical simulation of single neurons into easy-to-apply kernel representations that implicitly encode the collective statistics of relevant neuronal populations regarding axonal arbor geometry and distribution of synapses. This technique can be used to build up libraries of activation functions for neuronal populations that can define inputs to computationally efficient methods.*
- *Compared to Yu and colleagues (2024), our model does not attempt to solve the same problem (i.e. explaining the generation of cortico-spinal waves). Instead, we provide a model that links the electric field and particularly its direction to the elicitation of action potentials in presynaptic axonal arbors and further to the generation of postsynaptic currents. So, in a sense, our framework could be used to enhance the Yu model (which assumes random statistical activation of presynaptic axonal hillocks and afferents in connection with orthodromic propagation) by intracortical inputs motivated by our coupling model, which would allow for directionally sensitive simulations.*

The central point of introducing a realistic relationship between the electric field, action potential generation and propagation, and synapto-dendritic activation of downstream cells is elucidated in the "background" section. In addition, we added a clarifying paragraph to the discussion section:

Line 619 - 624
"In conclusion, the proposed framework provides a generic tool to couple TMS induced electric fields to dendritic currents entering the somata of cortical output neurons, thus determining their firing patterns. As many existing, otherwise sophisticated, models of the generation of short-latency cortical responses to TMS (i.e, DI waves) assume a very simplified relationship between action potential elicitation and electric field without any directional sensitivity (e.g., Rusu et al., 2014, Yu et al., 2024, Wilson et al., 2021), our framework offers a possibility to enhance these models without the need to carry out expensive single neuron simulations as in Aberra et al. (2018, 2020) and Weise et al. (2023)."

Reviewer #1: Other points that require some clarification are the following:

Reviewer #1: In Fig. 3B, does the time indicated in the caption (t = 0.525 ms) refer to the time of stimulation or is it the time of the frame?

Response: We changed the caption text in order to clarify that the time of the displayed frame is meant: "Single frame at t = 0.525 ms of axonal delay simulation from Video S1."

Reviewer #1: 1. Figure 4 would be more clear if the authors included some voltage traces corresponding to the different locations indicated with arrows and especially where the number of spikes is greater than one.

Response: Fig 4 and its caption were adjusted to include voltage traces of terminals whose action potentials arrive within a chosen bin, illustrating the time and spatial binning of action potential backpropagation delay:

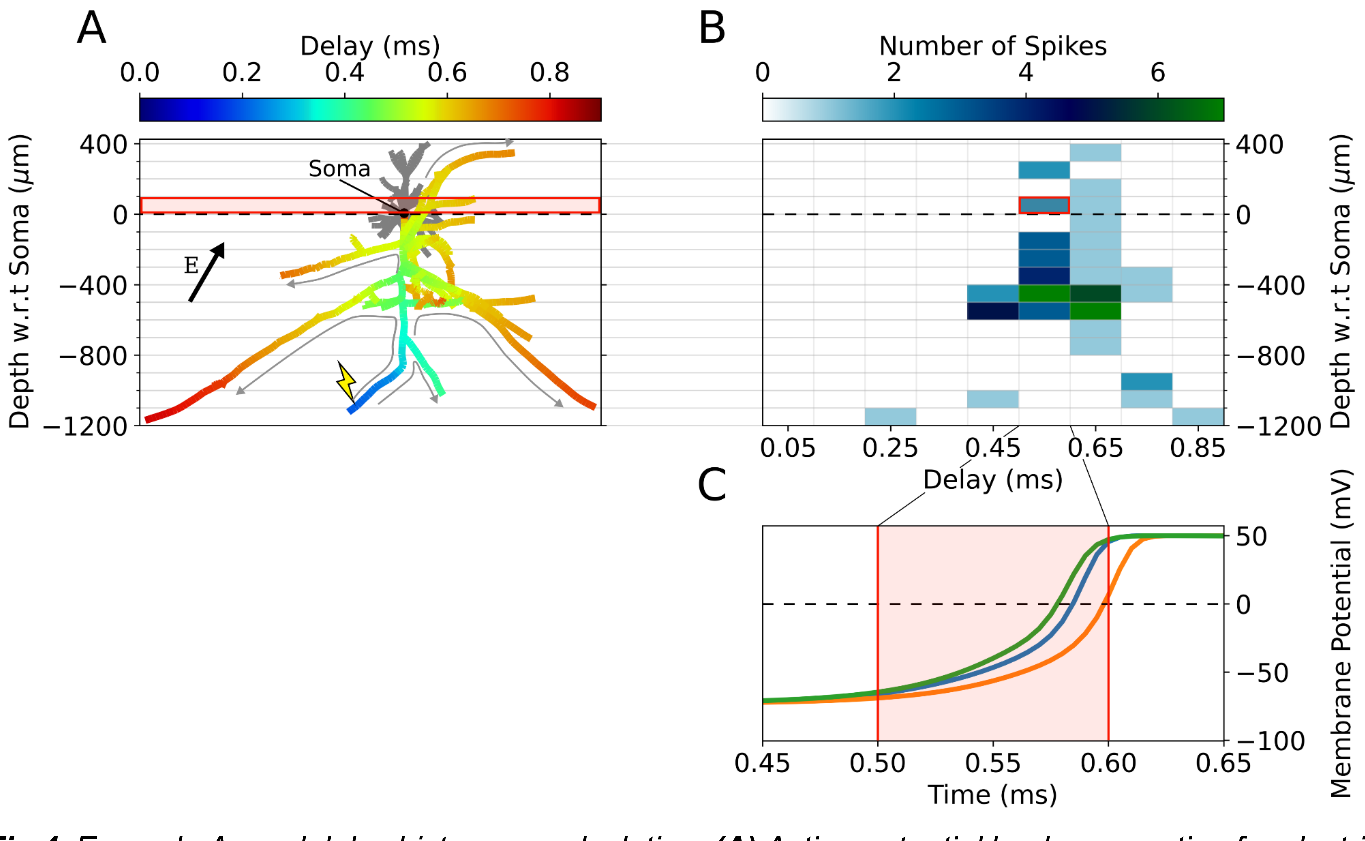

***Fig 4.** Example Axonal delay histogram calculation. **(A)** Action potential backpropagation for electric field parameters θ = 30°, Δ|E| = 0%, φ = 0°, |E| = 225 V/m, and biphasic pulse (Cell activation threshold is 140 V/m). Color axis indicates the arrival time of an action potential at the associated axon compartment in ms. Activation in this example occurs only at the terminal marked by the lightning bolt, with gray arrows depicting the backpropagation through the axonal arbor to other axon terminals. Boxed in red is an example z-bin between z = 0 µm and z = 100 µm. **(B)** Two-dimensional histogram of action potential arrival times and locations for each axon terminal in this cell. Boxed in red is a chosen histogram bin (with same z-values as in **(A)**) with depth between 0 and 100 µm and time between 0.5 and 0.6 ms. **(C)** Membrane potential timeseries for the three axon terminals whose action potentials arrive within the time-depth-bin boxed in **(B)** (i.e. their position lies within the depth bin and their potentials cross V = 0 mV within the time bin). The boundaries of the bin in the time axis are also boxed in red.*

Reviewer #1: 2. In line 234 they refer to Fig. 5A, B after the sentence “After averaging the axonal delay kernel histograms across each azimuthal angle, each cell morphology, and across cell subtypes for inhibitory basket cells”: however, Fig. 5 contains data related to pyramidal cells, and therefore I think that the sentence could be misleading. Please change it.

Response: We agree that this formulation can be misleading and changed it to:

Line 244 - 248
“After averaging the axonal delay kernel histograms across each azimuthal angle, each cell morphology, and across cell subtypes from Table 1, we end up with a histogram representing the average distribution of spike outputs in time and depth with respect to the soma. Fig 5 depicts the averaging processes for the case of L23 PCs, while this process is also carried out for L5 PC and L4 BC morphologies. Additionally, cells within a given layer are placed at a range of soma depths within the cortex.”

Reviewer #1: 3. One of the conclusions they draw from Fig. 9 (lines 423-425) is that “L23 PC axonal arbor geometry is slightly anisotropic, such that more axon terminals are oriented parallel to the somatodendritic axis”: is this the case? In other words, can the authors test independently the degree of anisotropy of L2/3 PCs?

Response: We are thankful to the reviewer for pointing this out. Indeed, this was just an intuitive interpretation that was not explicitly tested, and therefore it was by no means sure that simple directional statistics of the axonal terminals would explain the observed anisotropy. Indeed, when looking at the orientations of axonal arbors directly (see figure below), such an explanation cannot hold. Hence, there must be some, so far unknown, factor that makes L23 PCs more sensitive to electric fields parallel to the somatodendritic axis. As any explanation attempt appears very speculative at this moment, we decided to remove that claim from the manuscript.

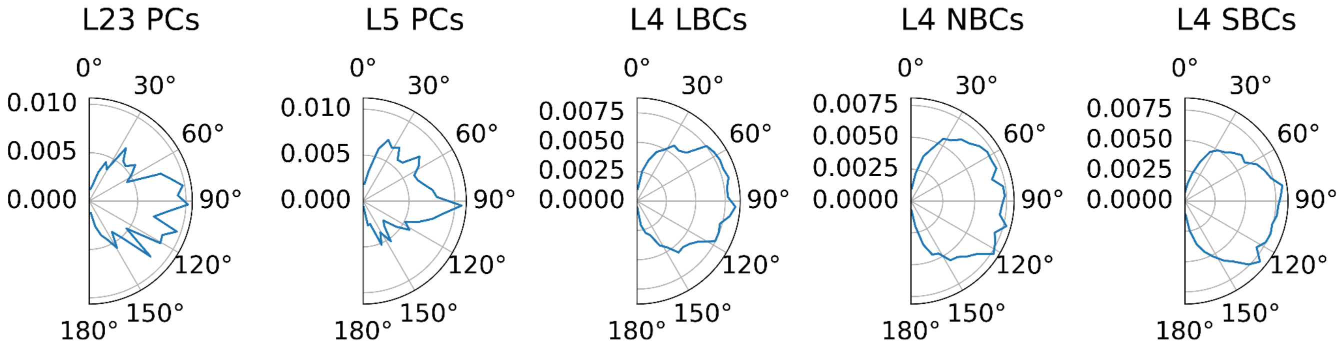

Figure: Histograms of mean orientation angle density of axonal terminals with respect to the axo-dendritic axis (perpendicular to cortical surface) for different cell types.

Reviewer #1: 4. The difference between z axis and z’ axis is not clear.

Response: The z coordinate denotes the distance from cortex-CSF boundary and points outwards (i.e., within the cortex, all z coordinates are negative), while z’ shares the same direction and orientation, but originates from the soma location of a particular neuron. In order to make this more clear in the text, we applied the following changes to the respective paragraph:

Line 259 - 272

"The population axonal delay kernel is computed numerically from the cell average as follows. Let the average spike density distribution with respect to the cell soma be represented by r(z', t). The variable z' notates position with respect to the cell soma in the axis parallel to the somatodendritic axis where z' ∊ [-2000 µm, +500 µm] in steps of 100 µm and total vector length N_r (Positive direction is oriented from the soma toward the dendrites. Exact ranges vary between morphological types due to cell sizes). z' = 0 µm lies at the cell soma. The cell density function d(z), however, is in the domain of the cortical depth axis (z-axis), where variable z represents position in the cortical column. z ∊ [-2700 µm, 0 µm] in steps of 100 µm and total vector length N_d. z=0 µm lies at the CSF boundary with layer 1, while z=-2700 µm lies at the layer 6 to white matter boundary at the bottom of the cortical column. The correlation of spike density distribution with the cell density function is executed only along the z-axis (which is parallel to the z'-axis) for every value of t. The time (t-axis) is unaffected by the correlation operation. SciPy's cross-correlation function (version 1.13.1) under the 'same' mode has an output the same length as the cell density function and lies in the same domain as the cortical depth axis (z-axis). The correlation process amounts to a change of basis from z' → z, i.e. from position with respect to cell soma to position with respect to cortical surface."

Reviewer #1: Minor notes:

- *Line 236: "within in" should be just "within".*
- *Lines 253-254, the values of z coordinate should be accompanied by the units of measure, e.g., z = -2700 um.*
- *In the caption of Fig. 6, there is a spurious "bf" before the label to panel (D).*
- *Line 472, "soma" should be "some".*
- *Line 479, "underlay" should probably be "underlie".*

Response: Thank you for pointing these out - we followed your advice in each case.

REFERENCES

[1] Aberra, A. S., Wang, B., Grill, W. M., & Peterchev, A. V. (2020). Simulation of transcranial magnetic stimulation in head model with morphologically-realistic cortical neurons. Brain stimulation, 13(1), 175-189.
[2] Yu, G. J., Ranieri, F., Di Lazzaro, V., Sommer, M. A., Peterchev, A. V., & Grill, W. M. (2024). Circuits and mechanisms for TMS-induced corticospinal waves: Connecting sensitivity analysis to the network graph. PLOS Computational Biology, 20(12), e1012640.

Reviewer #2: Reviewer summary

The authors present a methodology that acts as a surrogate to link action potentials generated by realistic electric field and neuron models to a transmembrane current that can serve as an input to other models, thereby bypassing the computational cost of simulating the realistic models. As a usage example, the authors applied this method to TMS activation of motor cortex and the generation of I-waves due to L2/3 activation, with applications toward coupling the activation to cortical network models.

The methodology is divided into two kernels. The axonal delay kernel empirically measures the spatio-temporal distribution of the spikes generated by coupling electric fields solved by the finite

element method to morphologically-realistic, compartmental models of neurons with Hodgkin-Huxley style dynamics. The synapto-dendritic delay kernel convolves the spike densities of the axonal delay kernel with the conductance impulse responses of AMPAR, NMDAR, GABAaR, and GABAbR models, and takes into account axial propagation, to generate a singular current that represents the combined synaptic effect, due to TMS activation, that a postsynaptic soma would experience. By computing these currents for each neuron in a population of neurons, a more realistic coupling of TMS-induced electric fields to a network can be achieved.

In general, this methodology is an important step toward addressing a gap in the field, that is to realistically couple the suprathreshold effects of TMS to neuronal network populations. The methodology has an initial large computational cost to generate the axonal delay kernels, but the author's plans to publish the kernels will alleviate the computational burden and serve to be an accessible tool.

However, there are three major concerns concerning the work. A line-numbered series of questions/comments follow these concerns.

Reviewer comments
Reviewer #1: 1. The quasi-1D cable equation that determines the amount of current arriving at the soma does not include the capacitive current. To properly represent the passive propagation from dendrite to soma, the capacitive current needs to be included.

Response: The neuronal model implemented in NEURON comprises the cable formulations of the dendritic and axonal sections, which includes the effects of membrane capacitance, as well as all relevant channel mechanisms. Therefore, in our opinion, the model does include the relevant capacitive currents.
We are, however, unsure whether we might not have fully understood the point of the reviewer.
In order to make this point clearer to the reader we added additional detail to the Neuron Models section:

Line 192 - 205
"In the NEURON simulation environment (version 3.9.19) interfaced via Python (version 3.9.19), morphologies were modeled as quasi one-dimensional cables with the inclusion of both active and passive biophysical properties including extended Hodgkin-Huxley-like ion channel models based on human neurophysiology. The morphologies are broken into straight sections of cell tissue coupled together, with each section broken into smaller compartments called segments. Segments serve as the discrete computational units whose membrane potentials are simulated and coupled to neighboring segments by capacitive, resistive, and synaptic trans-membrane and axial currents. Electromagnetic fields induced by brain stimulation generate a local extracellular pseudo-potential (Wang et al. 2018), which couples to the cable equations of individual neuron compartment models by integrating the electric field component along the axial direction of each compartment (Weise et al. 2023). Hodgkin-Huxley dynamics and cable theory drive action potential generation under electric field application and the propagation of action potentials on the axonal arbors and dendritic trees of these compartment models. For more detail on the modeled biophysical parameters, neuron morphology generation, and synapses see (Aberra et al. 2018)."

Reviewer #2: 2. In general, the section describing the construction of the synapto-dendritic delay kernels needs some clarification. It is not clear from the text that the measurements were made

directly in the L5 PC model. It is not clear that the kernel representations of the synaptic conductances were embedded throughout the morphology of the model, and simulations were run to measure the currents arriving at the soma. My initial interpretation was that the kernels were distributed according to the coordinates of the morphology but were not in actual mechanisms within the membrane, and the quasi-1D cable equation would only account for the passive properties of the dendrites. Rather, because the currents are propagated through the full model, the output current accounts for the passive and active properties of the dendrites and the morphological structure. This was only apparent after going through the github code. This section needs to be rewritten to make this more clear. Some of the line-numbered comments fall under this theme.

Response: We understand that the construction of the synapto-dendritic delay kernel was not so clear, so we have written additional description in the Coupling Pre- and Postsynaptic Simulations section to provide an overview of the model:

Line 296 - 311
"As TMS induced action potentials backpropagate through the axonal arbor of a cell, these action potentials inevitably reach axon terminals and synapse to other cells. To model the current input that a postsynaptic soma receives due to lying downstream of TMS activated cells, the inputs to the postsynaptic cell are determined by the axonal delay kernels of the upstream populations.

Foremost, for each set of electric field parameters, the axonal delay kernels of the L23 PC and BC populations determine the spatial-temporal distribution and magnitude of synaptic inputs to L5 dendrites. We then model these synaptic inputs as continuous conductance functions for four synapse receptor types directly computed from the axonal delay kernels embedded at each compartment on the L5 dendritic tree. The resulting dynamics is simulated to model the current entering the soma due to passive and active properties of postsynaptic potential evolution as well as dendrite geometry. The synapto-dendritic current is directly measured in-silico as the axial current at the interface between L5 PC dendrites and the soma. This simulation process is repeated and averaged across 30 different L5 PC morphologies as well as 25 L5 cell positions to characterize the average input received by all of L5 from L23."

Reviewer #2: 3. Because the method is an approximation of coupling of the finite element derived electric field to a neuronal population, there should be some characterization of how well the full model is approximated. How well does the axonal delay kernel capture the spatio-temporal distribution of spikes generated by a full finite-element / population model? When a L5 PC neuron is driven by the synapto-dendritic delay kernel output, how does its somatic membrane voltage differ from the case when its actual synapses are driven by the axonal delay kernel's spike densities (current injection from synapto-dendritic delay kernel vs synaptic activation due to suprathreshold axon terminals)?

Response: The axonal delay kernel does indeed directly capture the spatio-temporal distribution of spikes generated by a full finite-element/population model. The distribution is directly measured on morphologies. The resulting look-up tables are not subjected to any approximative function fitting or the like (the gPC approximation is only used for the sensitivity analysis).
Hence, the kernel is an approximation only in the sense that the underlying simulations are approximations. Its validity rests on the representativeness of the used axonal geometries, the validity of the used soma location distributions, among other assumptions.

The same applies to the synapto-dendritic kernel: the dendritic current distributions, as e.g. shown in Fig. 12, take into account the exact simulations of all the synaptic currents (of course averaged over morphologies, azimuthal angles, and cell positions).

Reviewer #2: Fig 4: Please add axis labels on left subplot.

Response: We added the labels (see new Fig. 4 above).

Reviewer #2: Lines 215, 302-303: The axonal delay kernel accounts for the z-position of the arrival/initiation of the action potential relative to the soma. Based on how the axonal delay kernel is linked to the synapto-dendritic delay kernel, it appears that the positions of the x- and y-positions of axon terminals relative to the x- and y-positions of the dendrites are not taken into account, e.g. regardless of the x- and y-distances of the dendritic compartment relative to the axon terminal, the latency of synaptic activation for dendritic compartments in the same z-location were identical. How much would including x- and y-distances impact the temporal properties of the axonal delay kernel?

Response: This is a good point. We actually assumed homogeneity of the axonal delay kernel in the x-y plane (e.g., the spike density depends on z only). While this seems a plausible assumption, at least in a statistical sense, we should mention this as a limitation in the Discussion section. Therefore, we adjusted the third-last paragraph of the Discussion section accordingly:

Line 588 - 596
"With respect to the synapse distribution algorithm, the synaptic coupling computed here defines synaptic inputs to the downstream population based on geometric overlap with axon terminals from the upstream population. In addition, we assumed that the axonal delay kernel is homogeneous within the x-y plane, i.e. that synaptic connections between layer 23 and layer 5 are random and uniformly distributed in the x-y plane. Some optical studies exist which estimate the spatial distribution of synaptic inputs, for example from L23 excitatory cells to L5 PCs in M1 [44]. However, no such data is available for the projection of L23 inhibitory cells to L5 PCs. As a result of this lack of data, and the desire to make the coupling model more independent of requiring detailed physiological experimental data, the synaptic coupling was rather formed intrinsically based on cell geometry and position."

Reviewer #2: Line 277: Please provide a brief description of the pchip interpolation method.

Response: We improved the description and added the following statements:

Line 444 - 447
"The axonal delay kernels for L23 PCs are reported below, where each is computed from histograms with bin sizes dz = 100 um and dt = 0.1 ms. They are interpolated using the piecewise cubic hermite interpolating polynomial method (pchip) (Fritsch et al. 1984) to dz = 1 um and dt = 0.005 ms. The pchip interpolator preserves monotonicity in the interpolation data and prevents overshoot if the data is not smooth."

Reviewer #2: Line 287: Which L5 PC morphology was used to extract the synapto-dendritic delay kernel?

Response: We simulated all PC morphologies as the downstream receiving cell, and then the dendritic current was averaged across all morphology instances. Note that in Fig. 6, we depict an example based on a single representative neuronal morphology. We acknowledge that the formulation in the text was not clear and modified the text accordingly

Line 309 - 311
"Fig 6 depicts an exemplar individual synapto-dendritic delay simulation, which models synaptic inputs and resulting postsynaptic potential dynamics on a single representative L5 PC placed at the average L5 soma depth."

The Fig 6 caption title was also changed to:

"Synaptic inputs and model output of a single example synapto-dendritic delay simulation."

(See also the line changes above for Line 296 - 311 which additionally clarify the synapto-dendritic delay kernel averaging process across multiple L5 morphologies).

Reviewer #2: Fig 6G and H: How are the synaptic conductances converted into a current? Where does the voltage in Eq 2 come from?

Response: The synaptic conductances are converted into currents via Ohm's law as described in Eq 2 directly. The voltage is the membrane potential of the compartment, i.e. the state variable that the simulation is updating each time step. The synaptic conductance for each synaptic receptor type was computed ahead of time by the convolution of the biexponential kernel function for that receptor with the crosscut of the axonal delay kernel (Eq 3 - 8). Thus, at each time step, each compartment receives four values of conductance input based on the axonal delay kernels for AMPA, NMDA, GABAa, and GABAb respectively. Eq 2 converts each conductance into synaptic current by multiplying the conductance by the membrane potential (shifted by each receptor's reversal potential to set the sign of the current). This can be confirmed by unit analysis on Eq 2 in that Ω^{-1} Volts = Amperes. The description of Eq 2 is adjusted as shown below:*

Line 317 - 320
"Trans-membrane currents are computed from conductance via Ohm's law for conductance based synapses:

$I_{mem} = g\ (v - E_{reversal})$

where g is the synaptic conductance, v is the membrane potential of the compartment (simulated state variable), and $E_{reversal}$ is the reversal potential of the synapse."

Reviewer #2: Line 295: How were the parameters in Table 2 determined? Were they chosen to fit EPSC properties such as peak current, time-to-peak, half-height width, etc?

Response: The synaptic parameters in Table 2 were adopted from experimentally constrained modeling studies that use the same mechanistic framework of dendritic synaptic integration that we employ here.
In particular, our synaptic kinetics (reversal potentials, and relative conductances of AMPA, NMDA, GABA_A, and GABA_B channels) follow the parameterization introduced in Rusu et al. (2014), who

modeled synaptic transmission from L2/3 neurons onto morphologically detailed L5 pyramidal neurons.
The rise and decay time constants were taken from the synaptic mechanisms used in Dura-Bernal et al. (2023), where they were not treated as free fitting parameters but were instead constrained by experimental synaptic physiology.
In that model, AMPA, NMDA, GABA_A, and GABA_B synapses were implemented using standard biophysical kinetic schemes whose time constants were taken from in-vitro electrophysiological measurements. These kinetics were selected to reproduce experimentally observed synaptic current rise times, decay times, and receptor-specific dynamics across cortical cell types.
We added a statement in the Methods section to make this clear:

Line 320 - 324
"Synaptic rise and decay time constants, reversal potentials, and receptor-specific kinetics were adopted from previously published biophysically detailed cortical models (Rusu et al., 2014; Dura-Bernal et al., 2023), where they were constrained by experimental electrophysiological measurements and shown to reproduce physiologically realistic EPSC and IPSC dynamics."

Reviewer #2: Line 302: "Each synaptic conductance convolves the double exponential kernel…" This should read "Each synaptic conductance is computed by convolving the double exponential kernel with the partial integral of…"

Response: The line was updated to match request and increase readability:

Line 331 - 333
"Each synaptic conductance (Eqs 3 - 6) is computed by convolving the double exponential kernels (Eq 7) and Fig 6F) with the partial integral of the axonal delay kernels across the bin of width Δz = 50 μm (Eq 8 and Fig 6D and E)."

Reviewer #2: Line 308: What were the total numbers of synapses that the L5 PC neurons had (AMPAR + NMDAR + GABAaR + GABAbR)? What discretization value was used for their morphologies (compartment size)? How does the choice of discretization value affect the final current output of the synapto-dendritic delay kernel? How were synapses distributed through the dendrites, e.g. evenly across all dendritic compartments or heterogeneously according to spine densities that can be dependent on the proximodistal location relative to soma?

Response: Synapses were not discretely distributed. Each compartment receives synaptic conductances for AMPAR, NMDAR, GABAaR and GABAbR directly computed from the axonal delay kernel, based on the position of the postsynaptic cell in the cortex (i.e. the position of each dendritic compartment). Thus, the input conductance is a function in time that depends on the positions of upstream axon terminals and when action potentials reach them. The net current reaching each compartment is a superposition of synaptic inputs from L23 PCs and L23 BCs. Initially while developing the model, we did in fact distribute synapses discretely and attempted to choose the discretization value based on experimental data for inputs to L5 dendrites. However, we discovered that the model was highly sensitive to the choice of discretization and that there is a lack of sufficient experimental data to adequately define synaptic discretization and distribution for excitatory and inhibitory inputs to L5 from L23. We decided instead to allow the spatio-temporal distribution of spike inputs to be directly defined by the axonal delay kernels themselves (therefore by the L23 PC and BC morphologies) and to compute the input current at each compartment as a

superposition of upstream inputs. The paragraph was supplemented with the following description to clarify:

Line 345 - 351
"By defining synaptic inputs in this way, each compartment receives net input conductances (i.e. current) for each receptor type, defined directly by the axonal delay kernels and thus the spatio-temporal distribution of action potential arrivals at upstream axon terminals. Synapses were not discretely distributed across the dendritic tree, rather, synaptic inputs were defined by the spike density function of axon terminals whose positions spatially overlap with each dendritic compartment. Thus, synaptic inputs to L5 were defined directly by the L23 morphologies and their response to TMS induced electric fields."

Reviewer #2: Line 317: What other voltage-gated ion channels were present in the models? Setting sodium conductances to zero would only eliminate sodium currents and only if the voltages of all compartments were at the equilibrium potential of the sodium channel. If there are other voltage-gated ion channels, then they will also contribute current to the soma if their conductances are not set to zero. The models should be simulated until the voltages of all compartments reach their steady-state. If the currents are non-zero at steady-state, then the non-zero value should be offset from the final kernel output.

Response: In addition to voltage gated sodium channels, other channels, including voltage gated potassium channels are also present on the soma and axons. These potassium channels do contribute current to the soma and are responsible for the secondary peak in the dendritic current output. However, this current is still significant and should be included in the simulation. Suppression of sodium channels serves only to suppress the firing of the cell, such that we can observe subthreshold dynamics. The intended purpose of our coupling model is to provide an input current to other models that simulate the firing dynamics of a L5 population. Therefore, as the dendritic current due to synaptic inputs flows to the soma, voltage gated potassium currents would continue to play a role in subthreshold current before, during, and after the sodium channels open and cell fires. We did however explore the background current and found that with zero synaptic inputs there is a 0.109 nA background current flowing into the soma. We have therefore subtracted this background from the final output and updated Fig 6 and Fig 12 accordingly as depicted below and included the following text in the paragraph describing Eq 9:

Line 366 - 369
"We found, however, that even at steady-state when synaptic inputs were not present, a 0.109 nA background dendritic current flowed into the soma. This background current was subtracted from the results to ensure we only represent current due to synaptic inputs."

In the Neuron Models section we added the following text to include how simulations were initialized in steady-state:

Line 191 - 192
"To ensure all simulations begin at steady-state, all simulations are first run for 10^{11} ms to allow the system to settle before any stimulus is applied."

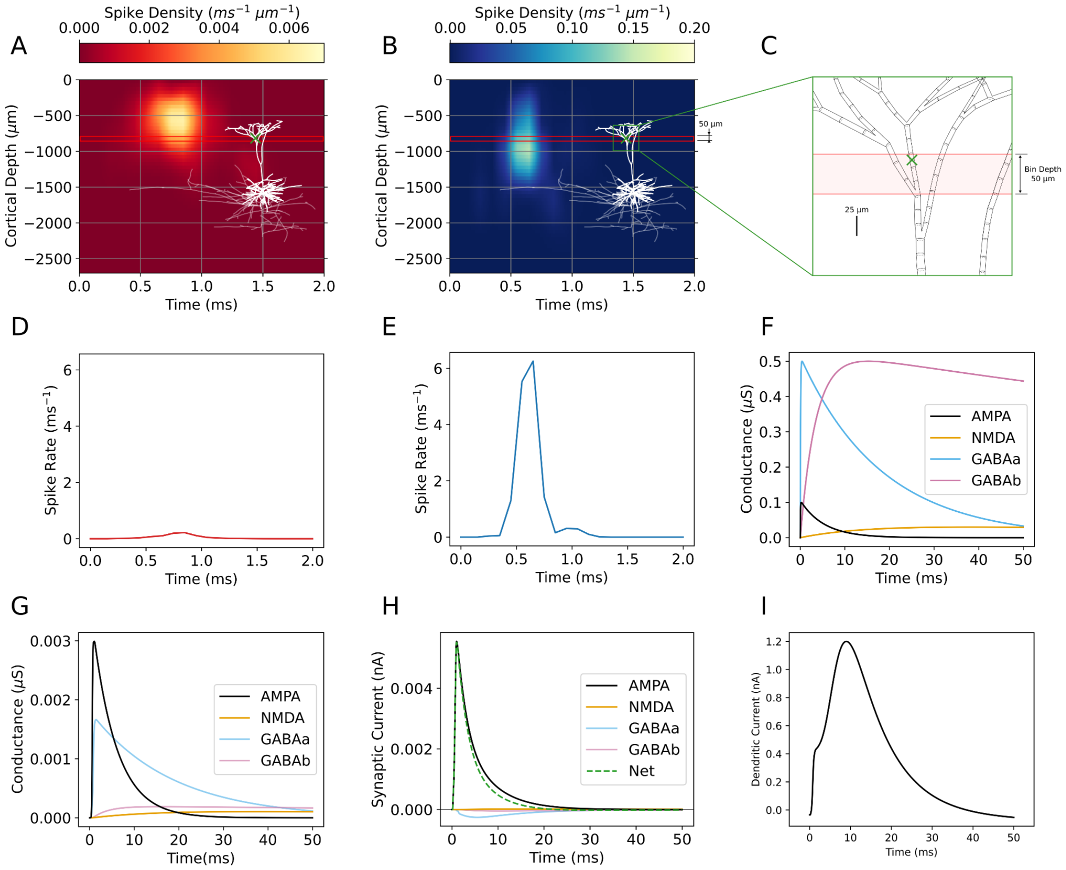

Updated Fig 6. (current shifted down by 0.109 nA)

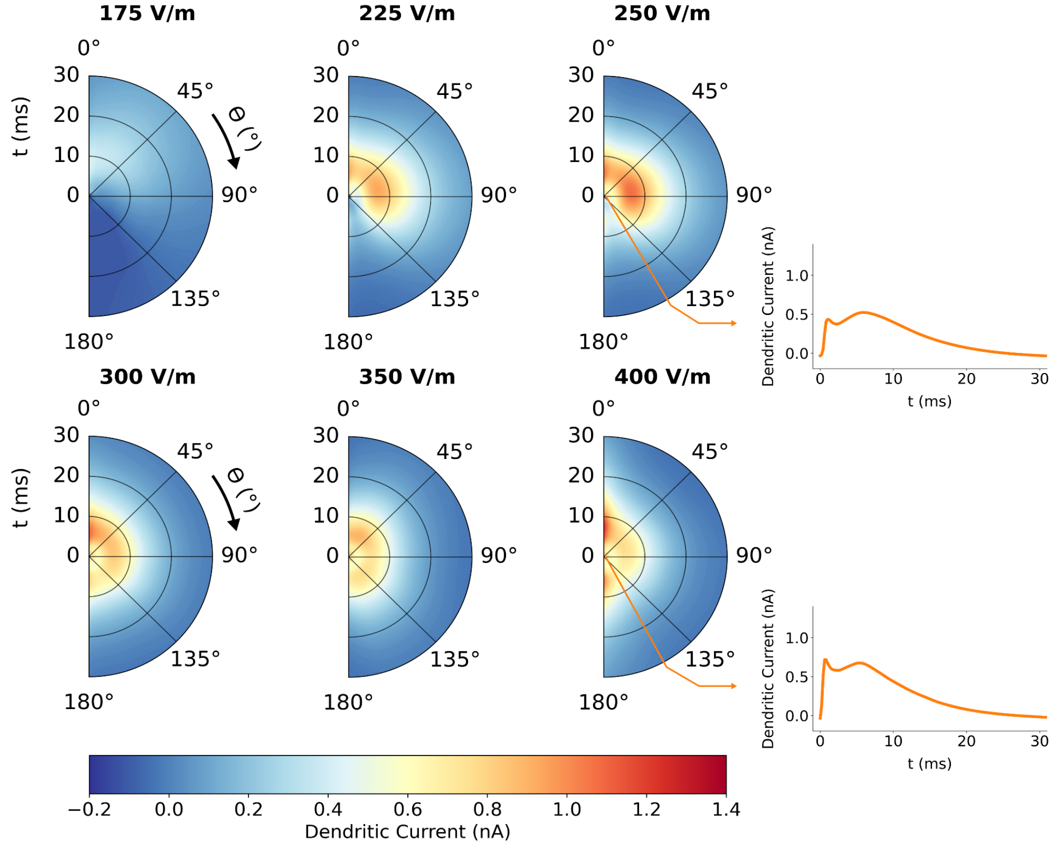

Updated Fig 12 (current shifted down by 0.109 nA)

Reviewer # 2: Lines 320–323; Eq 9: The synapto-dendritic delay kernel takes into account the effect of distance between the dendritic compartment and the somatic compartment using the quasi-1D cable equation but only takes into account the resistive current; the capacitive current is absent. To capture the total passive effect of the somato-dendritic integration, the capacitive current should also be included.

Response: Please refer to our response to your first comment. In our opinion, the cable equation implemented in NEURON captures both resistive and capacitive effects.

Reviewer #2: Line 324: "If there are N dendritic sections connected to the soma compartment, the current is calculated as the sum of contributions from each section with index i." Is the current calculated only from dendritic compartments directly connected to the soma, all dendritic compartments, or all dendritic compartments that have synapses? In the parlance of NEURON, are segments or sections being used to determine adjacency to the soma?

Response: As discussed in a previous comment, all dendritic compartments theoretically have synapses, although some of them may be receiving zero valued conductance if the input axonal delay kernels is zero at that depth. The current is calculated at the interface in between the soma and the initial dendritic sections. In terms of NEURON, we determine all dendritic sections which are children of the soma, i.e. the first dendritic sections which branch off of the soma. We calculate the potential gradient and thus the current at each of these initial dendritic sections such that the resultant sum of currents represents the net current flowing into the soma from the dendritic tree as a whole. See the diagram below which illustrates the calculation of dendritic current at the soma-dendrite interfaces including labeled variables as in Eq 9. The paragraph describing Eq 9 was also adjusted to:

Line 359 - 366

"If there are N dendritic sections directly connected to the soma, the current is calculated as the sum of contributions from each section with index i (Note that dendritic sections are distinct from segments in NEURON, where a section is a straight portion of morphology that may contain multiple segments). The membrane potential gradient for each adjacent dendritic section is calculated as the finite difference approximation between the potential at the soma, V_{soma}, and the potential at the center the of the dendritic section, V_i, divided by the path length distance from the center of the soma to the center of the adjacent section L_i. Each gradient is then scaled by cross-sectional area A_i over the axial resistivity R_i. The finite difference sign is chosen such that current flowing into the soma is positive."

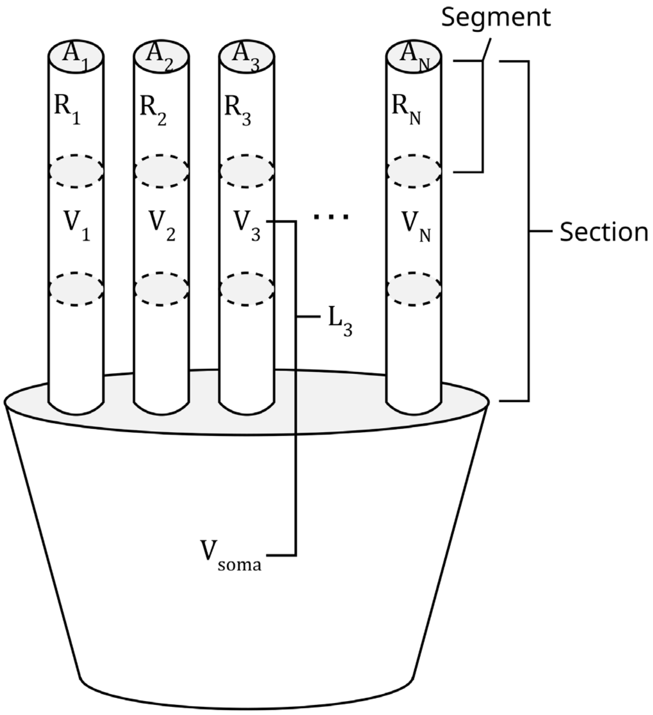

Reviewer #2: Lines 327-328: Is distance between dendritic compartment center and soma computed using the path distance along the morphology or the Euclidean distance between the coordinates?

Response: *Technically we calculated the path length distance (labeled L_i) along the morphology between the center of the first dendrite sections and the center of the soma. However, because each section object in the NEURON morphology is a straight line, the euclidean distance and the path distance end up being the same. We updated the description as seen in the previous comment:*

Line 361 - 365
"The membrane potential gradient for each adjacent dendritic section is calculated as the finite difference approximation between the potential at the soma, V_{soma}, and the potential at the center the of the dendritic section, V_i, divided by the path length distance from the center of the soma to the center of the adjacent section L_i."

Reviewer #2: Line 344: Please provide a brief description and/or general formula for the generalized polynomial chaos method.

Response: We improved the description of the surrogate model in Section "Surrogate Model of Dendritic Current" and added the general formula of the gPC approximation.

Line 395 - 417
"... The gPC is based on modeling the input-output characteristic of black box systems using an orthogonal polynomial basis. For time dependent problems, a separate gPC is performed for each of the time points. The gPC surrogate of the dendritic current then reads:

$$I(t,\xi) = \sum_{i=1}^{N_c} c_i(t)\, \Psi_i(\xi)$$

where the vector xi contains the free parameters described previously. For each time point, N_c gPC coefficients are determined independently.
The maximum order of the polynomials $\Psi_i(\xi)$ is chosen to ensure the lowest possible approximation error while still being computational feasible. The maximum individual order of the polynomials was set to 20 if only one parameter is present in the basis function, e.g. in case of the polynomial for angle θ, ξ_θ^{20}, except for ξ_Δ |E| describing the relative gradient of the electric field where the order could be reduced to six. The interaction order of the parameters ξ was limited to three in the basis functions, allowing all possible parameter combinations of up to three between the six input parameters. If parameter combinations are present in the basis function, the maximum sum of all interacting polynomials was limited to 8 in order to reduce the number of underlying polynomials in the six dimensional space. The time axis t was resampled to a time step of Δt=0.2 ms in the range of 0 and 100 ms. This resulted in N_c = 1642 polynomial coefficients to be determined for each of the N_t = 500 time points after resampling. In order to determine the corresponding polynomial coefficients, N_{sim} = 4000 simulations were conducted with random parameter combinations within the specified parameter limits and the time curves of the dendritic currents were saved for each parameter combination.
Summarizing all simulations, the gPC problem from Eq (11) can be formulated for all time points in matrix-form as:

$$[I] = [\Psi][C]]$$

…"

Reviewer #2: Lines 358–361: What does it mean that the maximum order of individual polynomials was set to 20? Can certain time points have a different polynomial order than others? What does it mean that maximum sum of all interacting polynomials was limited to 8? Please clarify how the orders of the polynomials were determined.

Response: We completely agree that the description of the polynomial orders is confusing for the general readership because there are many possible settings. We improved the explanation of the individual polynomial settings (see paragraph cited above). In short, if only one parameter is present in the basis function, its order can go up to 20 e.g. ξ_θ^{20}. If parameter combinations are considered, we allowed all possible three combinations between the parameters, which is in general enough in the framework of surrogate modeling to describe more than 99% of the variance. If multiple parameters are included in the particular basis function then the sum of all orders is limited to 8, e.g. $\xi_\theta^4\,\xi_E^2\,\xi_{AMPA}^2$.

Reviewer #2: Lines 455–456: Can the surrogate gPC model for the synapto-dendritic kernel be separated to compare the sensitivity analyses for L2/3 PC based (excitatory) activation vs L2/3 BC based (inhibitory) activation? Do these cell types exhibit different variances in dendritic current and directional sensitivities? Do different basket cell classes exhibit different properties as well?

Response: In our sensitivity analysis, the balance between excitatory and inhibitory input is captured by the parameter f_{ex}. This parameter represents the fraction of excitatory synaptic drive and therefore allows us to study the effect of varying E/I balance on the dendritic input current. For physiological realism, we restricted its range to [0.2, 0.8], to focus the analysis on the biologically relevant regime of mixed synaptic drive.

A decomposition into purely excitatory (L2/3 PC–driven) and purely inhibitory (L2/3 BC–driven) contributions is not possible for the synapto-dendritic kernel. This is because dendritic integration in our model is nonlinear, and therefore the principle of superposition does not apply: the combined response to excitatory and inhibitory input cannot be expressed as the sum of their individual responses. As a result, separate gPC surrogates for excitatory and inhibitory dendritic contributions would not yield the total input current.
In contrast, such a separation is feasible for the axonal delay kernel, where linear superposition holds, and excitatory and inhibitory effects can therefore be analyzed independently.

Reviewer #2: Fig 12: What contributes to the two peaks in the dendritic current? There is a sharper peak around 1 ms and a second broader peak around 6 ms.

Response: Thank you for pointing out this interesting observation, which is indeed worth a deeper look. We systematically quenched different mechanisms in the neuronal model and found that Shaw-related potassium channels (SKv3_1) are responsible for this effect (see figure below). We made a note about this in the Synapto-dendritic Delay Kernel Results section:

Line 499 - 502
"Note that the secondary peak visible in the dendritic current timeseries was found to be a direct consequence of Shaw-related potassium channels on the soma and axons (by systematic silencing of channels), also appearing at the single simulation level."

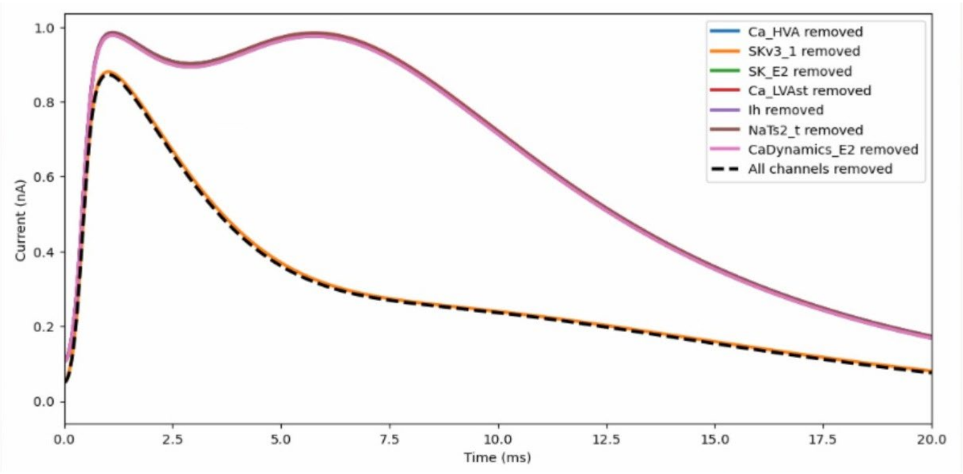

Reviewer #2: The amplitude of the dendritic current remains large up to 10 ms. Given that typical I-wave intervals can be 1-1.5 ms, this sustained current could potentially trigger 6-10 I-waves could occur during this time window. However, 6-10 I-waves are rarely observed in patients. What contributes to this discrepancy?

Response: The window with high dendritic currents only constitutes the maximum range for I waves to occur. There are multiple possible mechanisms that may limit the number of actually occurring waves, including desynchronization between cells due to variable refractory periods and early network effects through, e.g., inhibitory feedback. Modeling these mechanisms is beyond the scope of this paper. As stated before, we see our model as a mechanism that might complement other frameworks that are dedicated to the explicit elicitation of cortical-spinal waves (e.g., Yu et al., 2024). In addition, we are also conducting research of our own into that direction.

Reviewer #2: Line 523: Another limitation to include is that the method is strictly feedforward. Any action potentials that are generated during the injection of current produced by the synapto-dendritic delay kernel by the postsynaptic neuron will backpropagate through the dendrites and should affect the state of the synapses by modifying the membrane voltage, changing the state of the voltage-gated ion channels, etc. However, the present form of the methodology does not take into account the state of the neuron as the current drives the model (feedback).

Response: We thank the reviewer for highlighting this important limitation. We agree that, in principle, action potentials generated by the postsynaptic neuron during synapto-dendritic current injection can backpropagate into the dendrites and influence synaptic efficacy through changes in membrane voltage and the state of voltage-gated ion channels. Such feedback effects are not included in the present formulation of our method.

Our framework is intentionally restricted to a feedforward description in order to construct a tractable and interpretable synapto-dendritic delay kernel that links presynaptic population activity to postsynaptic dendritic input. Incorporating full feedback would require modeling spike-triggered dendritic nonlinearities, voltage-dependent synaptic dynamics, and recurrent population interactions, which would substantially increase model complexity and dimensionality. Moreover, the relevant feedback loops operate on timescales that are typically longer than the dominant synapto-dendritic integration window captured by our kernel. For example, a spike generated in an L5 pyramidal neuron must backpropagate into the dendrites, influence synaptic or interneuron activity, and then return via inhibitory or excitatory pathways to the same dendritic tree. Even assuming fast conduction and minimal synaptic delays, this loop introduces delays on the order of several milliseconds, which limits its influence on the short-latency synaptic current shaping that is the focus of our analysis.

We have added this point as an explicit limitation in the revised manuscript and note that extending the framework to include activity-dependent feedback and recurrent interactions is an important direction for future work:

Line 600 - 607

“The present framework provides a feedforward mapping from presynaptic population activity to synapto-dendritic input current and does not include activity-dependent feedback from the postsynaptic neuron. Action potentials generated during synaptic input backpropagate into the dendritic tree, modulate membrane voltage, and alter the state of voltage-gated ion channels and synaptic conductances, thereby influencing subsequent synaptic integration. These effects introduce nonlinear, state-dependent feedback that is not captured by the current synapto-dendritic delay kernel. Incorporating state-dependent feedback and recurrent dynamics would be necessary to capture spike-triggered dendritic nonlinearities and long-timescale network effects and represents an important direction for future extensions of the method.”
